# Evaluation of Quality Indicators of Integrated Care in a Regional Psychiatry Budget – A Pre-Post Comparison by Secondary Data Analysis

**DOI:** 10.5334/ijic.2479

**Published:** 2016-12-31

**Authors:** Anne Berghöfer, Svenja Hubmann, Thomas Birker, Torsten Hejnal, Felix Fischer

**Affiliations:** 1 Institute for Social Medicine, Epidemiology and Health Economics, Charité – Universitätsmedizin Berlin, Germany; 2 Clinic for Pediatrics, Klinikum Westbrandenburg, Brandenburg, Germany; 3 Clinic for Psychiatry, Psychotherapy, and Psychosomatics, Westküstenkliniken Brunsbüttel und Heide gGmbH, Heide, Germany; 4 Institute for Social Medicine, Epidemiology and Health Economics and Department of Psychosomatic Medicine, Center for Internal Medicine and Dermatology, Charité – Universitätsmedizin Berlin, Germany

**Keywords:** regional psychiatry budget, integrated care, quality indicator, secondary data analysis

## Abstract

The Regional Psychiatry Budget (RPB), as a special arrangement within the German Federal Hospital Refund Regulation, is based on the capitation principle. A lump sum is allocated to a major inpatient care provider in a large region on a yearly basis. Under this model, the provider is free to offer all forms of treatment and to construct individual models of integrated care that specifically suit the region and the needs of community members.

The present study aimed to evaluate selected aspects that represent a change in the psychiatric health status of patients in the covered region under the conditions of the RPB.

We performed a secondary data analysis of administrative data of 19,913 cases generated by the hospital in a pre-post comparison of the periods before and under RPB conditions.

The average length of an inpatient stay was reduced by approximately 22 % and could be partially replaced by day care. Selected indicators suggest equal or higher quality of care with stable cost in the population in need of psychiatric care in the district.

## Introduction

The treatment of chronic psychiatric disorders is seriously hampered by the division of the German health care system into the sectors of outpatient and inpatient acute care, rehabilitation and social support care [1,2]. Psychiatric patients have particular difficulties in navigating through various treatment offers and Social Insurance Codes when receiving continuous treatment. Case management is not routinely provided in the German health care system [3,4].

Under a major health system reform in Germany in 2000, the legislative authorities allowed several innovative models of care [5]. These models aim to overcome sector divisions and to provide continuous treatment, which integrates several care providers and guarantees the stability of treatment staff. Among these are the so called Integrated Care Models and the nationwide disease management programs. A third form of these innovative integrated models is the Regional Psychiatry Budget (RPB), according to paragraph 26 of the former Federal Hospital Refund Regulation (Bundespflegesatzverordnung), which is based on the capitation principle. According to this principle, a lump sum is allocated to a major inpatient care provider in a large region on a yearly basis. The negotiated lump sum is kept stable over the time span of the contract between the umbrella organisation of all statutory health insurance companies and the provider. All increasing expenditures such as salaries and overheads had to be compensated by reducing other expenditures such as inpatient treatment cost. In return the provider is free to offer all forms of treatment and to construct individual models of integrated care within the RPB that specifically suit the region and the needs of community members. The provider does not need to itemise services and will not be supervised by the medical review board of the statutory health insurance companies [6,7].

Despite the obvious advantages of this system for the provider’s discretionary power to deliver care, only a few regions in Germany have implemented a RPB so far. The majority are located in Schleswig-Holstein, where about one million inhabitants in six administrative districts are covered by a RPB. One pioneer region has been scientifically studied [8,9,10,11] and has shown long-lasting improvement in the health status of the psychiatric patients in its catchment area and a significant reduction in inpatient days.

The district of Dithmarschen began using the capitation principle only in 2008. Psychiatric and psychotherapeutic inpatient care for the approximately 135,000 residents of this predominantly rural district are provided by the only hospital that exists in the region. The hospital is committed to a social psychiatric treatment concept and provides psychiatric care by avoiding inpatient stays and offering treatment in day care facilities and walk-in clinics; this is combined with home treatment if necessary as well as long-term social support services and continuous therapeutic relationships. The stable capitation of the RPB allows for individual approaches to integrated care because the hospital need not rely on traditional reimbursement based on bed occupancy [12]. The model has been described in detail elsewhere [7,9,10,12].

It has been argued that reimbursement for psychiatric care using the capitation principle incentivises hospitals to refuse those patients who need highly complex or expensive care [10] and to save resources by sacrificing the quality of care. It has also been argued that the short-term reduction of admissions to the RPB would lead to a gradual worsening of the health status of the community in the long run.

The present study aims to evaluate selected aspects that represent a change in the psychiatric health status of patients in the Dithmarschen region who were included in the RPB. Using administrative data generated by the hospital and the basic patient documentation of the psychiatric department, the analysis of selected quality indicators should clarify whether the introduction of a RPB changes the treatment results of patients.

## Methods

### Study Design

**Figure 1:**
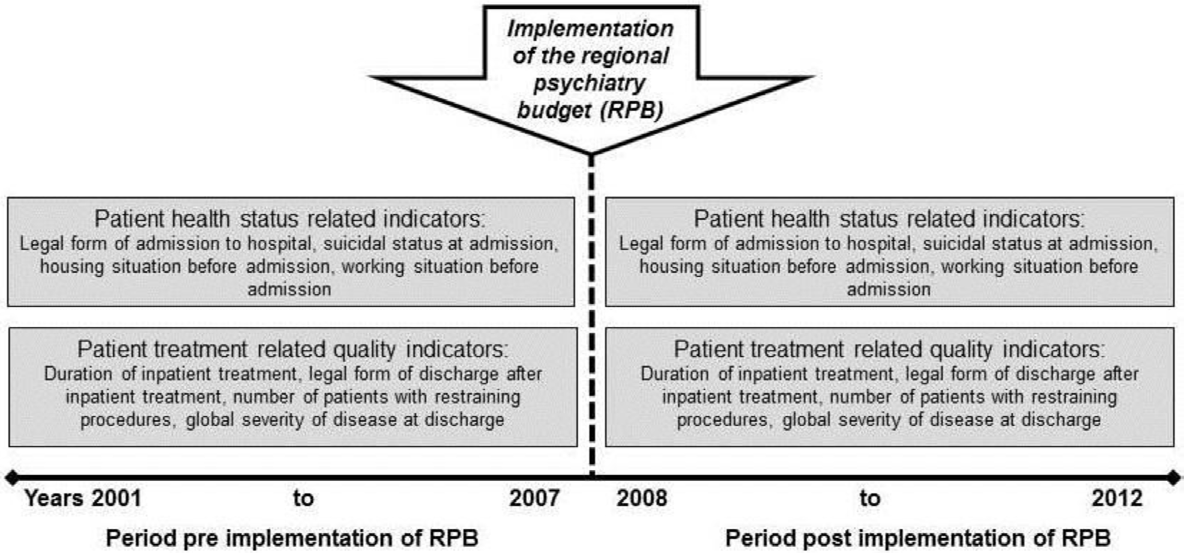
Pre-post comparison study design for evaluation of selected indicators of health status and quality of psychiatric care.

The study used a pre-post comparison design. All documented cases who received psychiatric care at the regional hospital during the years 2001–2007 (before implementation of the RPB) were compared to all documented cases who received psychiatric care at the regional hospital during the years 2008–2012 under capitation principle conditions within the Regional Psychiatry Budget (RPB) in the administrative district of Dithmarschen Figure 1.

### Selection of Indicators

We selected indicators of health status and social situations of the patients in the district as well as quality indicators of psychiatric care provided during hospital or day care stays. Indicators were eligible, if they were documented in both study periods (before and after implementation of RPB) and in addition were most robust against fluctuations of documentation quality and fluctuations of staff over the long observation period. Indicators were

– Legal form of admission to hospital as indicator of severity of illness
– Suicidality at time of admission as indicator of severity of illness
– Working situation before admission as indicator of social functioning and autonomy
– Housing situation before admission as indicator of social functioning and autonomy
– Duration of inpatient treatment
– Legal form of discharge after hospital or day care treatment
– Number of cases for whom restraining procedures were necessary during inpatient stay
– Global estimation of treatment response at time of discharge, based on the most recent psychiatric assessment by the treating physician and documented in the standardized documentation system.

The mean number of cases per year did not differ between the period before and after implementation of the RPB, as it is an inherent part of the contract between care providers and statutory health insurances, and providers have to cover the needs of their catchment area. Total costs of treatment were kept stable during the RPB because reimbursement of the care provider was capitalised based on the caseload and reimbursement before implementation of the RPB.

### Data Sources

Administrative, anonymised data for analysis were contributed a) by the hospital controlling department, which provided the number of cases admitted to inpatient or day care treatment per year, starting in 2001; and b) by the psychiatric department, which provided the number of forced admissions, the number of restraining procedures during inpatient stays (which must be documented in Germany), and the clinical parameters that were documented in a standardised basic psychiatric documentation system (BADO) for the admission and discharge of each case [13,14]. BADO data recording was performed in a Microsoft Access-based software system (PSYQUADO, [15]) between 2001 and 2007 and in a Microsoft Excel system between 2008 and 2012.

### Data Management

Data management and analysis were performed according to the Good Practice Secondary Data Analysis (GPS) of the Working Group for the Survey and Utilisation of Secondary Data (AGENS) of the German Society for Social Medicine and Prevention (DGSMP) [16]. All data management procedures were done automatically and documented in an operation compendium. Inpatient cases that were treated during both periods (including the date Dec 31, 2007) were artificially assigned to the pre-test period according to their admission dates.

### Statistical Analysis

Statistical analysis was performed using SPSS 22.0 for Windows [17] and R 3.0.2 [18]. Sociodemographic and treatment-related categorical variables were averaged over the years without and with the RPB; then chi^2^-Tests were performed with the null hypothesis that the distribution of the categories of variables was the same in both periods. We calculated 95%-confidence intervals for mean duration of stay assuming a normal distribution. The significance level for all analyses was set to 0.05. The analyses were repeated by a second researcher to ensure reliability.

### Ethical and Legal Considerations

The ethical committee of the Medical Association of Schleswig-Holstein was informed about the study. Because only anonymised data were used for analysis a formal ethical consultation was not required. Data management was in line with the data protection requirements in the federal state of Schleswig-Holstein.

## Results

A total of 19,913 cases could be included in the analysis. The mean number of cases per year remained approximately stable, with an average number of 1,659 (range: 1,534 to 1,799). The age at hospital admission was 46.6 before as well as after the RPB (p = 0.95). After introduction of the RPB the ratio of women increased from 45.8% to 49.4% (p < 0.001). While during the period before the RPB all cases were treated as inpatients, between 2008 and 2012 6,347 inpatient cases and 1,856 day care cases were documented. First line psychiatric diagnoses according to ICD 10 were organic, including symptomatic, mental disorders (F00–F09, 9.5%), mental and behavioural disorders due to psychoactive substance use (F10–F19, 37.8%), schizophrenia, schizotypal and delusional disorders (F20–F29, 14.8%), affective disorders (F30–F39, 14.7%), neurotic, stress-related and somatoform disorders (F40–F48, 13.5%), and other mental disorders (F50–F99, 9.7%). The distribution of first line diagnoses among these main categories remained stable between 2001 and 2012.

Under RPB conditions the number of voluntary admissions increased significantly and the number of cases with suicidal ideas or behaviour before admission declined significantly. The number of cases living in their own home increased and the number of cases in sheltered housing situations decreased significantly Table 1.

**Table 1:** Sociodemographic and clinical indicators of health status and social adjustment of psychiatric cases at time of admission before and after implementation of the Regional Psychiatry Budget (RPB) in the administrative District of Dithmarschen. P-values by Pearson’s Chi-Square, significance applies to the whole category values.

Indicator	Years 2001–2007	Years 2008–2012	p-values

Legal form of admission (n per year, %)			<0.001
Voluntary	1,527.7(92.6)	1,566.8 (95.4)	
Compulsory admission	86.0 (5.2)	50.8 (3.1)	
Prompted by legal guardian	36.6 (2.2)	18.6 (1.1)	
Other (e.g., underage)	0 (0)	5.8 (0.4)	
Suicidality at time of admission (n per year, %)			<0.001
Not suicidal	1,399.3 (84.7)	1,537.6(93.2)	
Suicidal ideas	193.9 (11.7)	69.0 (4.2)	
Suicide attempt	58.9 (3.6)	42.8 (2.6)	
Housing situation at admission (n per year, %)			<0.001
Living in own home	1,319.1(80.3)	1,416.6 (87.1)	
Assisted living	293.6(17.9)	188.2 (11.6)	
Homeless	29.0 (1.8)	21.8 (1.3)	
Working situation at admission (n per year, %)			<0.001
Full time employment	223.6 (14.5)	269.0(22.9)	
Part time employment	58.7 (3.8)	40.4 (3.4)	
Sheltered employment	104.1 (6.8)	45.4 (3.9)	
Stay-at-home partner	95.9 (6.2)	38.6 (3.3)	
Unemployed	465.7(30.3)	333.8(28.4)	
Retired	468.1(30.5)	337.2(28.7)	
In education	59.7 (3.9)	61.8 (5.3)	
Other	61.0 (4.0)	49.4 (4.2)	

The fraction of cases who responded to treatment and were discharged as improved increased significantly under the RPB. The number of cases needing any restraining procedures during their inpatient stays decreased significantly. Additionally, under the RPB cases were less likely to leave the hospital against medical advice or escape Table 2.

**Table 2:** Sociodemographic and clinical indicators of quality of psychiatric care of cases at discharge from hospital or day care before and after implementation of the Regional Psychiatry Budget (RPB) in the administrative District of Dithmarschen. P-values by Pearson’s Chi-Square.

Indicator	Years 2001–2007	Years 2008–2012	p-values

Legal form of discharge (n per year, %)			<0.001
Regular	1,318.7(79.8)	1,426.2(90.2)	
Against physician’s advice	267.4(16.2)	143.4 (9.1)	
Escaped	60.7 (3.7)	9.4 (0.6)	
Deceased	5.1 (0.3)	2.6 (0.2)	
Cases with restraining procedures necessary during inpatient stay (n per year, %)			<0.001
Yes	103.6 (6.3)	52.2 (3.2)	
No	1,544.6 (93.7)	1,582.0 (96.8)	
Global estimation of response at discharge based on psychiatric assessment (n per year, %)			<0.001
Improved	1,364.3(82.9)	1,501.0 (92.3)	
Unchanged	247.0(15.0)	91.6 (5.6)	
Worsened	34.3 (2.1)	33.8 (2.1)	

**Figure 2:**
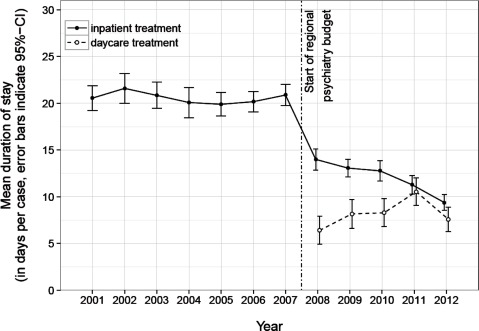
Mean duration of inpatient stay before and after implementation (dashed line) of the Regional Psychiatry Budget (RPB) in the administrative District of Dithmarschen.

The average length of an inpatient stay was reduced by approximately 22 % and could be partially replaced by day care Figure 2.

## Discussion

Under the capitation principle of a Regional Psychiatry Budget, the mean duration of inpatient treatment of psychiatric cases could be significantly reduced and shifted to day care and outpatient settings. Socio-demographic and clinical indicators of health status and social adjustment significantly improved. During hospital treatment, fewer restraining procedures were necessary and more cases could be regularly discharged, a higher percentage of those were improved compared to regular reimbursement conditions.

### Limitations

As a control group is missing, there are several limitations associated with the study design and data sources. As we performed a pre-post comparison, there are several threats to the internal validity of the analysis.

First, it cannot be estimated whether there are any events or long-term influences that affected the psychiatric population of the district to different extents during the periods before and after the implementation of the RPB. For example, the employment situation in Germany has generally improved during the last decade [19]. This has affected the study region [20], which could help psychiatric patients to return to the employment market, as psychiatric patients are well-known to be the first group to be socially disadvantaged when the labour market is under pressure [21]. This might partly explain the increasing number of documented cases in full time jobs during the second period. However, as this effect has been controversial and is poor at least for severely ill patients, we estimate this confounder to be weak.

Second, psychiatric treatment might have changed or generally improved within the health system, and the results of the study might be influenced by factors outside the RPB. Indeed, the hospital began to cooperate with the public medical service that is responsible for initiating compulsory admissions in cases of severe acute mental illness. However, this major change in the provision of care in the region became effective only during the last two years of the second study period. Additionally, it does not explain why other indicators changed between the first and second periods. Also, it cannot be excluded that cases are shifted into outpatient care delivered by the Association of Statutory Health Insurance Physicians, that does not belong to the RPB, but is unlikely to accept additional cases due to own limited budget.

Third, it is possible that the results might be biased by a change in data management between the pre and post observation periods. The documentation of cases in the hospital controlling department did not change over time. Only the documentation of basic patient and treatment characteristics in the psychiatric department changed between the periods, in terms of the software used and also the documented parameters and documenting persons. Additionally, the department tended to reduce documentation in general, as data verification by the medical review board of the statutory health insurance companies was stopped under RPB conditions.

However, in our analysis we did not use continuous variables or psychopathological parameters, both of which are sensitive to any methodological variations. We used only categorical variables, which were documented using standard operation procedures by the staff, and which we expect to be more robust against any changes in the data management process.

Finally, the data do not allow for a differentiated evaluation of psychopathological factors in the psychiatric population of the region. Detailed psychopathological documentation from the psychiatric department was not used for analysis, as parameters and their categories changed over time, as did the validity of documentation. Only the most robust parameters were analysed in this study. Additionally, we did not analyse subgroups of various psychiatric diagnoses. Therefore, it cannot be estimated whether individual diagnostic subgroups differ in their benefits from the RPB.

### Strengths of the Study Methods

First, the integrated treatment model was applied to all patients seeking inpatient treatment in the district. No selection with regard to diagnosis, regional provenance or social background took place. Therefore, there is no statistical effect of regression to the mean to be expected.

Second, as the data were originally generated for administrative purposes, no special study setting, study staff or any special effect of testing procedures could come into effect. The study question was set up only during a secondary data analysis. Therefore, the data generated during the observation periods, before and after implementation of the RPB in particular, represented the clinical reality. However, the results need to be replicated in other regions in Germany or in other health systems.

### Interpretation of results

In consideration of these limitations, no indicators of health status and social functioning of psychiatric patients in the region indicate any worsening associated with the implementation of the RPB in the long run, nor did indicators of quality of psychiatric care under conditions of the RPB indicate any worsening. Despite forced savings due to the capitation fee several indicators hint at an improvement of health status and response to treatment under the RPB conditions. Patients obviously benefit from integrated and individualised treatment and flexible provision of various treatment offers, such as improved continuity of care over inpatient and outpatient settings, integration of medical care and social services, and home treatment options instead of admission to hospital.

The results are in line with data from the RPB in the adjacent district [9,11], where costs for inpatient treatment were lowered and functioning of patients with schizophrenia and affective disorders improved significantly under the capitation principle.

## Conclusion

Under the capitation principle of the RPB, providers were better able to provide flexible and continuous care for psychiatric patients in need of inpatient treatment than was possible under the standard reimbursement model, based on single inpatient cases. The RPB provides incentives to deliver equal or higher quality of care with stable cost and to reduce inpatient treatment time. This did not result in a worse outcome in terms of health status and social functioning in the population of severely psychiatric ill patients in the region. The RPB enables healthcare providers to limit expenses. Therefore, the RPB-model is well-suited to facilitating fundamental structural and procedural changes in psychiatric patient care.
